# Dangers of Anæsthesia

**Published:** 1881-01

**Authors:** 


### DANGERS OF ANESTHESIA.

The *Cincinnati Lancet* considers that the condition of artificial anesthesia, no matter by what agent produced, is always a condition of danger. The term is somewhat vague, too much so, indeed, for strict reasoning. We may say that travel on a railroad always constitutes a condition of danger. We agree with the *Lancet*, however, in the general expression. The truth is that anesthesia is really partial death. It is a suspension of one of the most important functions of life. Although we have witnessed the condition with countless frequency during all the long years since the introduction of ether, yet we must confess that every instance of anesthesia still brings to mind the idea of death, and produces the impression that the petient is pushed so near to the grave as to suggest the possibility that he will not return.

